# Exploring Unmet Needs Related to Cow’s Milk Protein Allergy Among Pregnant Women and Mothers of Infants and Young Children: A Qualitative Study

**DOI:** 10.3390/nu18132178

**Published:** 2026-07-04

**Authors:** Zoe Harbottle, Brenna Morton, Brianna Hunt, Jennifer L. P. Protudjer, Kristin A. Reynolds

**Affiliations:** 1 Department of Pediatrics and Child Health, Rady Faculty of Health Sciences, Max Rady College of Medicine, University of Manitoba, Winnipeg, MB R3A 1S1, Canadajennifer.protudjer@umanitoba.ca (J.L.P.P.); 2 Children’s Hospital Research Institute of Manitoba, Winnipeg, MB R3E 3P4, Canada; bhunt@chrim.ca; 3 Department of Psychology, University of Manitoba, Winnipeg, MB R3T 2N2, Canada; 4 Institute of Environmental Medicine, Karolinska Institutet, 17177 Stockholm, Sweden; 5 Department of Food and Human Nutritional Sciences, University of Manitoba, Winnipeg, MB R3T 2N2, Canada; 6 Department of Psychiatry, University of Manitoba, Winnipeg, MB R3T 2N2, Canada

**Keywords:** cow’s milk protein allergy, maternal impacts, mental health, unmet needs, qualitative

## Abstract

**Background/Objectives:** Cow’s milk protein allergy (CMPA), one of the most common allergies in early life, can present psychological, financial, and social challenges for caregivers. For mothers, this burden may be compounded by the fact that experiences with pediatric CMPA typically coincide with the perinatal period, which itself may be challenging. Our study aimed to explore the concerns, experiences, and needs of mothers navigating pediatric CMPA. **Methods:** We conducted a qualitative study involving three groups: mothers of children (18 months to 4 years) with, or who previously had, CMPA; mothers of infants (<18 months) with CMPA; and pregnant women concerned about their baby developing CMPA. All mothers completed a demographic questionnaire and participated in a virtual focus group or interview that was recorded, transcribed verbatim, and analyzed according to reflexive thematic analysis. Questionnaire data were described (n/N, %) to summarize participant characteristics. **Results:** In total, 16 mothers participated. Three themes were identified: (1) perceived negative psychosocial impacts, (2) perceived inadequacy of support from healthcare professionals, and (3) perceived negative dietary and financial implications. **Conclusions:** This study provides valuable insights into the perceived psychosocial implications and unmet management needs of CMPA.

## 1. Introduction

Raising a child with a food allergy can present several challenges for parents, as notable lifestyle changes are required to accommodate the child [1]. Unsurprisingly, pediatric food allergy has been associated with negative outcomes for caregivers, including reduced quality of life, greater levels of anxiety and significantly higher food costs [2,3,4,5,6]. A limited number of studies provide evidence that parents of children with food allergy may experience symptoms of depression [2,7,8].

Navigating pediatric food allergy may be especially difficult for those in the perinatal period (pregnancy to 12 months postpartum) [9], as this period is linked with several biological, psychological, financial, and social changes that, independent of food allergy, can serve as sources of stress [10,11]. Some research supports that pregnant women tend to report concerns about their baby developing a food allergy, which may be further exacerbated if an immediate family member has a food allergy [12]. The stress associated with the perinatal period, coupled with concerns about food allergy and the stress of caring for an infant with food allergy, may leave mothers especially susceptible to developing symptoms of anxiety and depression. Indeed, even without accounting for factors such as child allergy, psychopathology, particularly anxiety and depression, is common during the perinatal period [9]. The estimated prevalence of anxiety disorders is around 15.5% during the prenatal period and ranges from 9.6% to 17.1% during the early postnatal period [13,14]. At 11.9%, the estimated prevalence of perinatal depression parallels that of perinatal anxiety [13,14]. Taken collectively, it is plausible that perinatal mothers parenting an infant with food allergy are at an even greater risk of experiencing symptoms of anxiety or depression.

While all food allergies demand considerable management, cow’s milk protein allergy (CMPA) management may pose challenges, particularly during the perinatal period [15,16]. Self/parent-reported prevalence estimates of CMPA range from 1.3% to 17%, with most cases emerging in the first year of life [17,18]. Historically, CMPA was thought to typically resolve by school age. However, recent evidence has suggested there is considerable variability in the rate of resolution [19]. While over half of young children with CMPA are likely to develop tolerance [20], almost half of children may experience persistence of CMPA in childhood [19].

Traditionally, CMPA management involved the total elimination of milk protein [21]. It remains debated whether mothers who are breastfeeding an infant with CMPA must eliminate milk protein from their diet [22]. While dietary elimination generally reduces symptoms, prolonged dietary restrictions may be associated with several nutritional risks for both mothers and children [22]. Further, the ubiquity of milk protein in food products makes CMPA the most burdensome food allergy, with accidental exposures resulting in severe adverse events [21,23]. Recently, there has been a shift towards treatments, including milk ladders and oral immunotherapy, which gradually reintroduce milk protein to promote tolerance [21]. These and other treatments offer hope, but are not consistently available or an appropriate option for all patients.

Although parents of infants face considerable burdens, few interventions specifically targeting mothers of children with CMPA have been investigated. Most existing interventions are educational in nature, and access is limited [24]. These limitations underscore the need to develop evidence-based psychosocial supports that are accessible to all. A first step in developing these supports is to better understand the lived experience and unmet needs of mothers whose children have CMPA. Therefore, we aimed to explore the concerns, experiences, and unmet needs of mothers navigating pediatric CMPA and the concerns and knowledge related to CMPA of pregnant mothers using a qualitative approach.

## 2. Materials and Methods

### 2.1. Participants and Recruitment

In this qualitative study, participants were recruited through online parent groups and social media posts. Respondents were then screened via a questionnaire hosted on Qualtrics. Participants were deemed eligible if they: (1) self-identified as a mother of an infant age <18 months with CMPA, a mother of a child (18 months to 4 years) who previously had or currently has CMPA, or a pregnant mother concerned that their infant may develop CMPA; (2) were aged 18+ years; (3) had stable internet access; (4) were comfortable reading, writing, and speaking in English; (5) did not have a diagnosis of a severe psychological disorder; and (6) had an infant/child with no chronic conditions other than allergy. To limit participant burden and account for variability within diagnostic processes, this study relied solely on parent-report of CMPA diagnosis.

### 2.2. Data Collection

**Figure 1:**
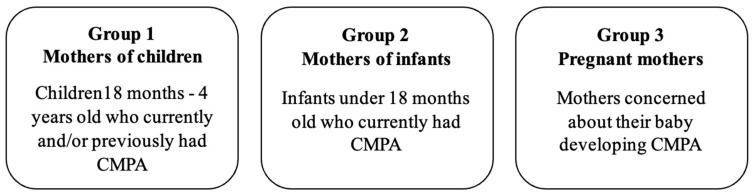
Summary of participant groups.

After informed consent was obtained, participants completed a 20 min background questionnaire specific to the group with which they self-identified. Thus, there were three versions of the questionnaire, i.e., one per group (Figure 1). Questionnaires included demographic variables, breastfeeding experiences, mental health history, allergy in self and children, thoughts and behaviours surrounding CMPA, and allergy literacy.

Following completion of the background questionnaire, participants took part in either a virtual focus group or an individual interview, per their preference and availability. Focus groups and interviews utilized group-specific semi-structured guides. Groups 1 and 2 were asked about their experiences with and impacts of CMPA, while participants in Group 3 were asked about their experiences with pregnancy and their concerns surrounding CMPA. Additionally, all participants were asked about their allergy literacy; resources they have accessed or are interested in accessing; preferred formats, types, and sources of support; and obstacles to accessing resources. Local mental health resources and contact information for crisis help lines were provided to all participants in screening questionnaires, consent forms, and email communications. Each participant was offered a $50 Canadian Dollar (CAD) e-gift card for their time.

### 2.3. Data Analysis

Descriptive statistics were used to quantify sociodemographic characteristics and symptoms of and treatment sought for CMPA.

Focus group/interview data was uploaded to Trint, a transcription software. Transcripts were then analyzed according to reflexive thematic analysis [25,26]. Reflexive thematic analysis occurred in the following phases: (1) familiarization with the data, (2) generation of initial codes, (3) identification of patterns within the data, (4) review of themes with co-authors, (5) definition and naming of themes, and (6) production of the report [25,26]. Each transcript was analyzed separately and then compared. Two researchers independently coded all data and developed preliminary themes. Analysts then met with co-authors to discuss the thematic framework and establish overarching themes. To ensure rigour, the researchers engaged in reflexive practice, critically reflecting on their positionality and its influence on research processes and findings [27,28].

## 3. Results

### 3.1. Participant Characteristics

In total, 16/21 mothers who consented and provided demographic data participated in a focus group or interview (Table 1). A total of four focus groups and four interviews were completed. Focus groups were on average 72 min (range 62–81 min) and interviews were on average 21 min (range 13–32 min). All participants identified as female, were married or in a common-law relationship, and had post-secondary education. Most (14/16, 87.5%) identified as having European ethnic or cultural origins.

Group 1 mothers (n = 6) were, on average, 33.7 ± 1.6 years old. Their children were, on average, 5.8 ± 2.2 months at CMPA diagnosis and 29.5 ± 11.5 months at interview. Half reported that their child no longer had CMPA, and half had previously followed a cow’s milk elimination diet.

Group 2 mothers (n = 6) were, on average, 32.2 ± 3.2 years old. Their infants were, on average, 3.0 ± 1.7 months at CMPA diagnosis and 9.8 ± 4.6 months at interview. All mothers indicated that they had previously or were currently following a cow’s milk elimination diet.

Participants in Group 3 (n = 4) were, on average, 32.5 ± 4.5 years old, and, at the time of interview, they were, on average, 24.6 ± 9.8 weeks pregnant. Half of the participants were slightly concerned about their baby developing CMPA, while the other half were somewhat or moderately concerned, respectively. Furthermore, half of the participants rated themselves as not at all knowledgeable about CMPA, whereas the other half rated themselves as slightly or somewhat knowledgeable, respectively.

### 3.2. Maternal Experiences Surrounding CMPA

We identified three themes: perceived negative psychosocial impacts; perceived inadequacy of support from healthcare professionals; and perceived negative dietary and financial implications (Table 2).

#### 3.2.1. Perceived Negative Psychosocial Impacts

For many participants, aspects of caring for a child with CMPA were perceived to be associated with negative psychosocial impacts. Namely, many expressed feeling scared and overwhelmed at the onset of symptoms. For example, participants, notably those with infants, who saw blood in their baby’s stool described feeling very afraid. Relatedly, some participants described a sense of guilt stemming from the perception of being at fault for their baby’s discomfort. One participant stated they felt like they had contributed to the severity of their child’s allergy, as they had kissed or touched their child after eating certain foods.

Furthermore, many participants reported feeling socially isolated and avoiding social gatherings due to a lack of understanding or accommodation among family and friends. Several participants with older children described family members who would try to accommodate; however, the ingredients they used for cooking contained cow’s milk. This was often associated with a misunderstanding of CMPA and, in some cases, confusion of CMPA with lactose intolerance. Another participant mentioned having a family member who did not believe allergies existed, making this individual unsafe for their child to be around. For some, this lack of understanding and accommodation also came from friends and childcare providers. For example, a participant described instances at their child’s daycare where other parents would bring in cow’s milk-containing treats, leading to their child ingesting the allergen. However, some participants expressed feeling supported by family or friends. Notably, one participant described attending a mom’s group where others would ask to ensure snacks were safe before bringing them. Nevertheless, most participants described some degree of limited social participation related to their child’s CMPA.

Many also described feeling anxious about social gatherings. Some participants noted that the stress of declining food or the additional effort required to bring their own meals led them to avoid these situations altogether. Additionally, some, typically mothers of older children, described anxiety related to concerns of their child feeling left out and the stress of possible accidental exposure when other children were consuming cow’s milk in social situations.

#### 3.2.2. Perceived Inadequacy of Support from Healthcare Professionals

When symptoms first presented, many participants sought help from a healthcare professional but felt that their concerns were dismissed. This was especially evident among participants whose first point of contact was their general practitioner (GP) or pediatrician. For example, one participant felt that their pediatrician dismissed their concerns and implied that they were overreacting. Another described how they could not access a pediatrician due to a shortage and felt as though they had to tell their GP what steps to take to receive adequate care. Notably, those who visited the emergency department at a children’s hospital generally shared positive experiences. However, many of these participants followed up with their GP or pediatrician afterwards and were dismissed or met with a lack of support.

Many participants requested a referral to see an allergist or gastrointestinal specialist. Participants often expressed having to push for a referral; those who did not generally did not receive one. Many shared their frustrations with wait times for specialists, as they felt that they had little guidance on how to manage their child’s symptoms in the meantime. Indeed, several participants expressed how helpful earlier intervention would have been for both managing symptoms and assuaging anxieties. Once seen by an allergist, participants described allergists as a helpful resource, particularly with guidance on reintroduction. However, one Group 2 participant noted that while their allergist seemed very knowledgeable about allergies, they lacked knowledge about breastfeeding; therefore, some suggestions were not realistic.

In response to the perceived lack of support from healthcare professionals, many participants sought additional information online. Participants searched for resources pertaining to symptom management, prognosis, milk-free recipes, and reintroduction (e.g., the milk ladder). Participants noted that some resources, such as moms’ groups on social media that shared dairy-free recipes, were helpful as they navigated CMPA management. However, participants also described a sense of information overload when seeking guidance online, which, in turn, increased their anxiety.

This need to seek out information amidst a lack of support from healthcare professionals was further compounded by the fact that many participants did not have much knowledge about CMPA prior to encountering their child’s symptoms. This initial lack of knowledge was especially evident among pregnant participants: those who had no prior experience with CMPA, had minimal knowledge about CMPA, and were not especially concerned about their baby developing it. Participants from Groups 1 and 2, however, indicated that they wished they had been better prepared, with some suggesting that it would be helpful for public health nurses to provide information on CMPA presentation.

#### 3.2.3. Perceived Negative Dietary and Financial Implications

For many, managing CMPA required dietary changes for both mother and baby. Several participants discussed struggles with cutting allergens out of their diet while breastfeeding. Specifically, many participants discussed anxiety surrounding accidentally ingesting cow’s milk and inflicting discomfort on their child, as they found cow’s milk to be a “hidden ingredient”. Some participants, particularly those whose infant refused dairy-free formula, discussed the pressure of feeling as though they were the sole provider of nutrition for their baby. As a result of dietary elimination, participants expressed feeling that both themselves and their baby were not receiving adequate nutrition. For example, a participant discussed feeling worried that their child’s drop off on the growth curve was due to them eliminating milk from their own diet. Additionally, participants shared that they felt dairy-free alternatives did not replace dairy in terms of their nutritional content. Others also expressed displeasure with the taste of some alternatives, adding that this made it difficult for them to get enough to eat. As participants navigated the elimination of cow’s milk and replacing it with alternatives, many expressed wanting access to a registered dietitian.

Managing CMPA also came with substantial financial costs related to switching to specialized formulas as well as finding dairy-free alternatives for participants who were breastfeeding and their children, if they had started solids. For example, one participant described how, if not for a prescription program that covered the cost of a specialized formula, they would not have been able to afford to feed their baby. Others noted that even items such as dairy-free granola bars and vegan cheeses were noticeably more expensive than their non-allergen-friendly counterparts. Many participants expressed a wish for more support to offset this increased financial cost. For example, one participant noted that a moms’ group distributed coupons for cow’s milk, but not for cow’s milk alternatives.

## 4. Discussion

In this qualitative study involving mothers of children who have/previously had CMPA, mothers of infants with CMPA, and pregnant mothers concerned about CMPA, we identified three themes, namely, perceived negative psychosocial impacts, perceived inadequacy of support from healthcare professionals, and perceived negative dietary and financial implications. Findings from this study provide important insights about the burden of CMPA, as well as a roadmap to craft impactful interventional programs to support mothers.

While most participants had limited knowledge about CMPA prior to their infant/child’s diagnosis, or during pregnancy, the type of information desired varied throughout the stages of child development. Pregnant mothers with no prior experience with CMPA knew little about, and had little concern about, their infant developing this condition. Yet, mothers whose infants/children had CMPA expressed wishing they had known more about CMPA before symptom presentation and diagnosis and had greater access to reliable information from their healthcare provider, while also emphasizing the information overload experienced through their own online searching.

Within this division between little concern and information overload exists a time when infants commonly receive their first foods. Since the publication of the landmark study, Learning Early about Peanut Allergy [29], infant feeding guidelines around the world were revised to shift from delayed to early introduction of all foods around ages 4–6 months to optimize primary prevention [30]. In 2024, Chan et al. published a review extending beyond early introduction that emphasized the importance of regular ingestion, rather than intermittent exposure, going so far as to say that the latter “*increases the risk of cow’s milk allergy*” [31]. A similar author group published a plain-language infographic intended to be shared by healthcare professionals with their patients [30]. This allergy-expert-developed resource should be considered a critical form of knowledge translation between provider and parent. Thus, considering early introduction guidelines and the shift from no concern to overwhelm from pregnancy to the perinatal period described in this and other studies [32,33,34], delaying such discussions until a mother is ready to introduce solids is likely too late.

As with other pediatric food allergies, CMPA can pose many difficulties for caregivers. Among parents of children with multiple food allergies, CMPA has been reported as carrying the greatest financial, time, emotional, and social burdens [6]. Particularly, concerns tied to CMPA, such as the risk of cross-contamination, the cost and difficulty of finding alternative energy and calcium sources, and the marketing of milk towards children, may serve as sources of parental stress [6]. Golding and colleagues implemented a CMPA-friendly food supplement program, finding that parents who received this intervention reported reduced time costs associated with their child’s CMPA, but not necessarily reduced financial costs [35]. Additionally, dietary and financial concerns were described by mothers prior to their infant starting solids. The cost and availability of specialized formulas and finding suitable dairy-free alternatives while following an elimination diet were notable concerns [36].

We acknowledge the limitations of this study. Our sample was relatively small and characterized by females in their 30s, mainly of European ethnic or cultural origins, who had completed post-secondary education, worked full-time, and were partnered. As such, our findings may not be transferable to other populations. That said, if well-resourced mothers of infants/children are experiencing psychological distress and overwhelm due to CMPA management as described by participants, there is a critical need to explore these constructs in more diverse populations. Also, with recruitment via social media posts and online parent groups, parents experiencing greater disease burden or those with higher levels of health literacy may have been more likely to respond. Finally, this study relied upon parent-reported CMPA diagnosis and did not differentiate between Immunoglobulin E (IgE)-mediated and non-IgE-mediated CMPA; therefore, the findings should be interpreted in light of possible variability in diagnostic pathways.

Looking to the future, early exposure to information on early introduction delivered by allergy-informed professionals is key to combatting both overwhelm and misinformation. As access to allergists is limited by a relatively low proportion of allergists per capita, the majority of whom are based in large urban centres, interdisciplinary allergy-expert-delivered training is critical for primary care providers and allied health professionals. Additionally, cognitive behavioural therapy has been shown to be effective in reducing anxiety in parents of children with food allergy [37,38,39]. Access to psychological support could be improved through e-health/telehealth treatment. Further, education-based interventions, such as food allergy handbooks [40], or consultations with knowledgeable healthcare professionals [41,42] can serve to increase knowledge, and, in turn, improve quality of life. Finally, to address the social isolation inherent to CMPA, peer-to-peer mentorship programs may be beneficial, wherein mothers with longer lived experience are trained to provide practical support and a listening ear to mothers of infants who have recently been diagnosed with CMPA.

## 5. Conclusions

In conclusion, this study described the perceived psychosocial implications of CMPA on mothers whose children have/previously had CMPA, mothers of infants with CMPA, and pregnant mothers concerned about CMPA. Additionally, this study provides valuable insight into the unmet needs of these mothers to support efforts aimed at improving resources available for families managing CMPA.

## Figures and Tables

**Table 1:** Demographic characteristics of participants by group.

Characteristic	Group 1 (n = 6)	Group 2 (n = 6)	Group 3 (n = 4)
Age of mother, mean (SD), years	33.7 (1.6)	32.2 (3.2)	32.5 (4.5)
Female, n (%)	6 (100.0)	6 (100.0)	4 (100.0)
Married, n (%)	5 (83.3)	4 (66.7)	3 (75.0)
European ancestry, n (%)	6 (100.0)	5 (83.3)	3 (75.0)
University education, n (%)	5 (83.3)	5 (83.3)	3 (75.0)
Full-time employment, n (%)	5 (83.3)	0	2 (50.0)
Child age at CMPA diagnosis, mean (SD), months	5.7 (2.2)	2.9 (1.8)	NA
Child age at time of study, mean (SD), months	29.2 (11.3)	9.7 (4.7)	NA
Weeks pregnant, mean (SD)	NA	NA	24.6 (9.8)

Abbreviations: CMPA, cow’s milk protein allergy; NA, not applicable; SD, standard deviation.

**Table 2:** Qualitative themes and supporting quotations.

Theme	Supporting Quotations
Perceived Negative Psychosocial Impacts	“Coming from me to him felt like a whole other complicated issue, because I’m supposed to be the one feeding him food that is safe for him, especially when it comes directly from my body.” (P1, Group 1) “Social things that used to feel fun, don’t feel fun because there’s so much work. Or scary that something might happen.” (P9, Group 2)
Perceived Inadequacy of Support from Healthcare Professionals	“I had a GP who basically I just went to and would tell him what I wanted because they would not give me much. Like had no idea, wouldn’t even ask me any questions when I was there was like, yeah, we’re fine here bye like, here’s her shots, time to go. So not really feeling like there was any, any support there.” (P4, Group 1) “I’m pretty, like, health literate and able to, like, peruse through this information carefully. But even then, I found myself like, just going into like, anxiety spirals by the reading things on the Internet. I think like especially on social media, like you can get like information overload, and it can kind of like make you think about the worst case scenario it can like just, yeah, it can be very toxic at the same time.” (P6, Group 1) “I have been reading a lot of books on like becoming a parent and stuff like that. Like, I have three books that I’m reading kind of simultaneously, and none of them really touch on this at all. So to be honest, it’s not even something that I like was super aware of or like put a lot of thought into because I feel like it’s not touched on in a lot of current, like parenting resources.” (P13, Group 3)
Perceived Negative Dietary and Financial Implications	“A place where I felt under-resourced was my own diet. When I was breastfeeding, having to be off dairy and soy, I found it really hard to get enough to eat. And because [my baby] was so little, like, life was just really chaotic, and it was hard to, to cook meals. And, yeah, I was just fatigued and kind of felt unwell a lot.” (P11, Group 2) “Our bills definitely have gone higher for groceries.” (P10, Group 2)

Abbreviation, P Participant.

## Data Availability

Data is not available due to the highly personal nature of qualitative data.

## Abbreviations

CMPA	Cow’s Milk Protein Allergy
GP	General Practitioner
IgE	Immunoglobulin E
